# Musculoskeletal radiologist-level performance by using deep learning for detection of scaphoid fractures on conventional multi-view radiographs of hand and wrist

**DOI:** 10.1007/s00330-022-09205-4

**Published:** 2022-11-15

**Authors:** Nils Hendrix, Ward Hendrix, Kees van Dijke, Bas Maresch, Mario Maas, Stijn Bollen, Alexander Scholtens, Milko de Jonge, Lee-Ling Sharon Ong, Bram van Ginneken, Matthieu Rutten

**Affiliations:** 1 Radiology Department, Jeroen Bosch Ziekenhuis, Henri Dunantstraat 1, 5223 GZ ‘s-Hertogenbosch, the Netherlands; 2 Jheronimus Academy of Data Science, Sint Janssingel 92, 5211 DA ‘s-Hertogenbosch, the Netherlands; 3 Department of Medical Imaging, Radboud University Medical Center, Geert Grooteplein Zuid 10, 6525 GA Nijmegen, the Netherlands; 4 Radiology Department, Noordwest Ziekenhuisgroep, Wilhelminalaan 12, 1815JD Alkmaar, the Netherlands; 5 Radiology Department, Ziekenhuis Gelderse Vallei, Willy Brandtlaan 10, 6717 RP Ede, the Netherlands; 6 Radiology and Nuclear Medicine Department, Academic Medical Center, Meibergdreef 9, 1105 AZ Amsterdam, the Netherlands; 7 Radiology Department, Groene Hart Ziekenhuis, Bleulandweg 10, 2803 HH Gouda, the Netherlands; 8 Radiology and Nuclear Medicine Department, Tergooi, Van Riebeeckweg 212, 1213 XZ Hilversum, the Netherlands; 9 Radiology Department, St. Antonius Ziekenhuis, Soestwetering 1, 3543 AZ Utrecht, the Netherlands; 10 Cognitive Science and Artificial Intelligence Department, Tilburg University, Warandelaan 2, 5037 AB Tilburg, the Netherlands

**Keywords:** Scaphoid bone, Fractures, bone, Artificial intelligence, Multicenter study, Clinical decision support system

## Abstract

**Objectives:**

To assess how an artificial intelligence (AI) algorithm performs against five experienced musculoskeletal radiologists in diagnosing scaphoid fractures and whether it aids their diagnosis on conventional multi-view radiographs.

**Methods:**

Four datasets of conventional hand, wrist, and scaphoid radiographs were retrospectively acquired at two hospitals (hospitals A and B). Dataset 1 (12,990 radiographs from 3353 patients, hospital A) and dataset 2 (1117 radiographs from 394 patients, hospital B) were used for training and testing a scaphoid localization and laterality classification component. Dataset 3 (4316 radiographs from 840 patients, hospital A) and dataset 4 (688 radiographs from 209 patients, hospital B) were used for training and testing the fracture detector. The algorithm was compared with the radiologists in an observer study. Evaluation metrics included sensitivity, specificity, positive predictive value (PPV), area under the characteristic operating curve (AUC), Cohen’s kappa coefficient (κ), fracture localization precision, and reading time.

**Results:**

The algorithm detected scaphoid fractures with a sensitivity of 72%, specificity of 93%, PPV of 81%, and AUC of 0.88. The AUC of the algorithm did not differ from each radiologist (0.87 [radiologists’ mean], *p* ≥ .05). AI assistance improved five out of ten pairs of inter-observer Cohen’s κ agreements (*p* < .05) and reduced reading time in four radiologists (*p* < .001), but did not improve other metrics in the majority of radiologists (*p* ≥ .05).

**Conclusions:**

The AI algorithm detects scaphoid fractures on conventional multi-view radiographs at the level of five experienced musculoskeletal radiologists and could significantly shorten their reading time.

**Key Points:**

*• An artificial intelligence algorithm automatically detects scaphoid fractures on conventional multi-view radiographs at the same level of five experienced musculoskeletal radiologists.*

*• There is preliminary evidence that automated scaphoid fracture detection can significantly shorten the reading time of musculoskeletal radiologists.*

**Supplementary Information:**

The online version contains supplementary material available at 10.1007/s00330-022-09205-4.

## Introduction

Scaphoid fractures, the most common type of carpal bone fracture (82–89%) [1], are typically diagnosed through clinical and conventional radiographic examination. However, it is well known that they can be difficult to detect on initial radiographs: reported percentages of missed scaphoid fractures vary from 7 to 50% [2–4]. If missed scaphoid fractures are left untreated and become displaced, the risk of developing non-union can be high (14–50%) [5]. Non-union fractures can have serious complications, such as progressive degeneration and collapse of the carpal bones [6]. Hence, more than half of the patients receive unnecessary wrist immobilization out of precaution [7, 8]. Since non-union fractures and overtreatment increase costs in healthcare and lost productivity, it is important to investigate strategies to aid early and accurate scaphoid fracture diagnoses.

In general, two diagnostic strategies have been discussed in the literature. The first strategy involves complementing or replacing initial conventional radiography with follow-up conventional radiography (10–14 days) or advanced imaging modalities such as CT and MRI. The second strategy involves utilizing the diagnostic value of conventional radiography via artificial intelligence (AI) software. Karl and Swart [9] and Yin et al [10] showed that immediate CT and MRI scans were more cost-effective strategies compared to follow-up radiographs except when lost productivity from immobilization is slight. However, if there is a limited number of scanners available or if the cost imposed to use these scanners is too high, conventional radiography is the primary or only means for imaging scaphoid fractures.

There is a growing body of literature demonstrating that deep learning–based AI software can obtain a diagnostic performance comparable to clinicians in detecting fractures at imaging [11]. Recently, Hendrix et al [12] and Yoon et al [13] respectively demonstrated that AI software can achieve radiologist-level performance on scaphoid fracture diagnosis in conventional radiographs and that it can detect occult fractures with high accuracy, thereby showing that AI has the potential to aid radiologists in detecting scaphoid fractures. Large-scale retrospective studies conducted by Duron et al [14] and Guermazi et al [15] showed that AI software could indeed improve the sensitivity and specificity of radiologists and other physicians in detecting various skeletal fractures. However, performance measures for scaphoid fracture diagnosis were lacking and only Duron et al let musculoskeletal (MSK) radiologists participate in their observer study, who are specialized in diagnosing skeletal fractures. Moreover, previous works [12, 13, 16, 17] on automated scaphoid fracture diagnosis only involved the use of anterior-posterior (AP) and posterior-anterior (PA) radiographs, whereas in clinical practice multiple radiographic views such as oblique and lateral views are used. These limitations raise the question whether previous findings hold when scaphoid fracture diagnosis is conducted with multi-view radiographs and whether AI software can improve the performance of radiologists in this setting, particularly that of MSK radiologists.

The purpose of this study was therefore to assess how an AI algorithm performs against experienced MSK radiologists in detecting scaphoid fractures on conventional multi-view radiographs and to assess whether it can aid MSK radiologists in clinical practice.

## Materials and methods

### Datasets

**Fig. 1:**
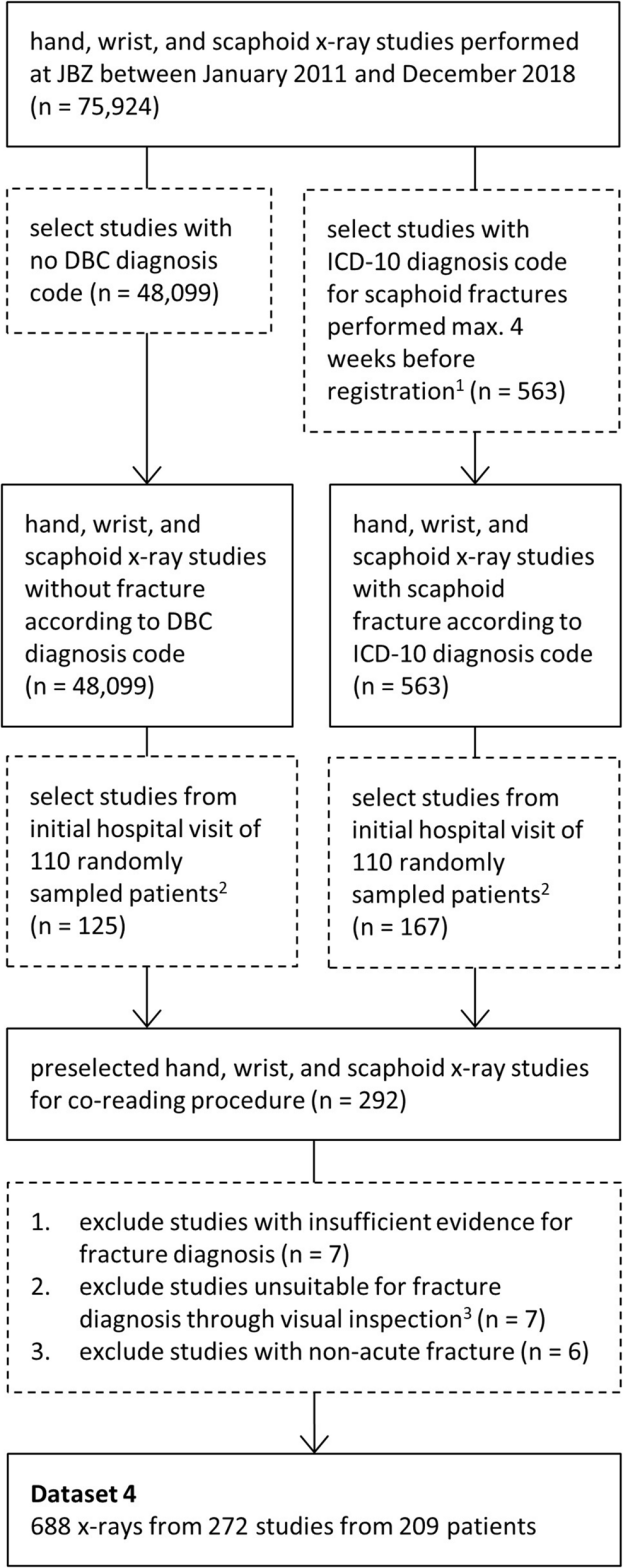
Flowchart for the inclusion and exclusion of samples in dataset 4 (test data). The number of studies at each step is denoted with n. DBC, diagnosis-treatment combination; ICD-10, International Classification of Diseases Version 10; JBZ, Jeroen Bosch Hospital. ^2^Studies with ICD-10 diagnosis code S62.00 were included. ^3^There was no patient overlap between the samples from the non-fracture and fracture category. ^4^Studies were excluded when the wrist was in cast, the scaphoid was incompletely depicted, or when there was severe scapholunate advanced collapse

This retrospective study was approved by the medical ethical review boards of the Radboud University Medical Center (Radboudumc) and the Jeroen Bosch Hospital (JBZ) in the Netherlands. Informed written consent was waived, and data collection and storage were performed in accordance with local guidelines. Dataset 1 (12,990 radiographs [from 3353 patients] acquired during 2003–2019 at Radboudumc) and dataset 2 (1117 radiographs [from 394 patients] acquired during 2018–2019 at JBZ ) served for respectively training and testing two auxiliary convolutional neural networks (CNNs) for scaphoid localization and laterality classification. Dataset 3 (4316 radiographs [from 840 patients] acquired during 2003–2019 at Radboudumc) and dataset 4 (688 radiographs [from 209 patients] acquired during 2011–2018 at JBZ) served for respectively training and testing a CNN-based fracture detection algorithm. The training and test datasets were gathered at different hospitals to assess the generalization performance of the algorithm. Only radiographs acquired at the initial hospital visit were included in dataset 4 as we focused on early fracture detection. Furthermore, the number of available radiographic views varied per study and patient. An overview of the characteristics of the datasets is provided in Table 1 (refer to Appendix E1 [online] for additional imaging parameters). This table also describes a patient overlap between these datasets and those from Hendrix et al [12]. Data from the previous study was added to the training datasets (datasets 1–3) to reduce the annotation effort, and ten patients (out of 209 [5%]) overlapped between the test dataset of the present study (dataset 4) and previous study as result from random sampling. The annotation protocol and the inclusion and exclusion criteria are provided in Appendix E2 and E3 (online). A flow chart of the study selection for dataset 4 is shown in Fig. 1.

**Table 1:** Details of the experimental datasets

Variable	Dataset 1	Dataset 2	Dataset 3	Dataset 4
Task	Train and validate scaphoid localizer and laterality classifier	Test scaphoid localizer and laterality classifier	Train and validate scaphoid fracture detector	Test scaphoid fracture detector
No. of patients	3353	394	840	209
Overlap with prior work^1^	289 (9.0%)	37 (9.0%)	686 (82.0%)	10 (5.0%)
Sex				
Male	1623 (48.4%)	140 (35.5%)	471 (56.1%)	102 (48.8%)
Female	1730 (51.6%)	254 (64.5%)	369 (43.9%)	107 (51.2%)
Age				
All	41 ± 22	44 ± 25	37 ± 20	39 ± 23
Male	37 ± 20	37 ± 24	33 ± 17	33 ± 21
Female	46 ± 22	48 ± 26	43 ± 22	45 ± 23
Number of radiographs	12,990	1117	4316	688
Radiograph location				
Hand	2229 (17.2%)	0 (0.0%)	327 (7.6%)	233 (33.9%)
Wrist	3922 (30.2%)	883 (79.1%)	1019 (23.6%)	280 (40.7%)
Scaphoid	6839 (52.6%)	234 (20.9%)	2970 (68.8%)	175 (25.4%)
Image size, pixels				
Hand height	1907 ± 476	NA	1991 ± 598	1632 ± 304
Hand width	1633 ± 472	NA	1691 ± 486	1015 ± 266
Wrist height	1644 ± 511	1226 ± 233	1768 ± 535	1284 ± 283
Wrist width	1353 ± 447	595 ± 122	1411 ± 461	624 ± 182
Scaphoid height	1190 ± 492	720 ± 238	1222 ± 499	886 ± 438
Scaphoid width	1373 ± 752	535 ± 132	1443 ± 769	627 ± 326
Pixel size, mm				
Hand	0.134 ± 0.019	NA	0.124 ± 0.022	0.143 ± 0.005
Wrist	0.129 ± 0.021	0.146 ± 0.002	0.123 ± 0.022	0.143 ± 0.004
Scaphoid	0.127 ± 0.021	0.146 ± 0.002	0.125 ± 0.021	0.142 ± 0.009
Number of scaphoids depicted^2^	15703	1117	5639	688
View				
AP/PA	7400 (47.1%)	492 (44.0%)	2497 (44.3%)	278 (40.4%)
Ulnar-deviated AP/PA	816 (5.2%)	68 (6.1%)	346 (6.1%)	57 (8.3%)
Oblique	3351 (21.3%)	116 (10.4%)	1302 (23.1%)	181 (26.3%)
Lateral	4136 (26.4%)	441 (39.5%)	1494 (26.5%)	172 (25.0%)
Statistical analysis				
Unit of analysis	Individual scaphoid	Individual scaphoid	Scaphoids grouped by study	Scaphoids grouped by patient
Count	15703	1117	1718	219
Fracture status				
With fracture	NA	NA	854	65
Without fracture	NA	NA	864	154
Annotation type(s)	- Scaphoid bounding box - Binary label (left/right scaphoid)	- Scaphoid bounding box - Binary label (left/right scaphoid)	- Multi-label (scaphoid fracture presence in six regions) - Binary label (left/right scaphoid)	Multi-label (scaphoid fracture presence in six regions)
Source(s)	Radboudumc	JBZ	Radboudumc	JBZ
Period	01/2003–04/2019	12/2018–03/2019	01/2003–04/2019	01/2011–12/2018

Note. Percentages with respect to the total dataset size are in parentheses. The mean image and pixel size, as well as the mean patient age, are reported with the standard deviation. Rounding errors were resolved using the largest remainder method. *JBZ*, Jeroen Bosch Hospital; *NA*, not applicable; *Radboudumc*, Radboud University Medical Center

^1^Overlap in patients from the scaphoid segmentation and fracture detection datasets in Hendrix et al (see ref 12). Datasets 1 and 2 were compared with the scaphoid segmentation training and test set, and datasets 3 and 4 were compared with the fracture detection training and test set

^2^Radiographs in the datasets (computed radiography [CR] and digital radiography [DR] images) can depict both hands of the patient or may show a compilation of multiple views of the same hand (in CR images only). Hence, the total number of depicted scaphoid bones is separately reported

### Ground truth

Two MSK radiologists (K.v.D. and M.R., with 22 and 26 years of experience, respectively) determined the ground truth for dataset 4. For each patient they determined the fracture presence in six scaphoid regions as defined by Wong and Ho [18]. These regions included the following: scaphoid tubercle (A1), distal articular (A2), distal 1/3 (B1), middle 1/3 (B2), proximal 1/3 (B3), and proximal pole (C). All cases were independently reviewed and disagreements were resolved by consensus reading. Both radiologists had access to all available imaging information (conventional radiography, CT, and MRI studies) and clinical information (clinical questions, and patient demographics and history) in the PACS and electronic health record (EHR) system (refer to Appendix E4 [online] for an overview of the reference standards).

### AI pipeline

**Fig. 2:**
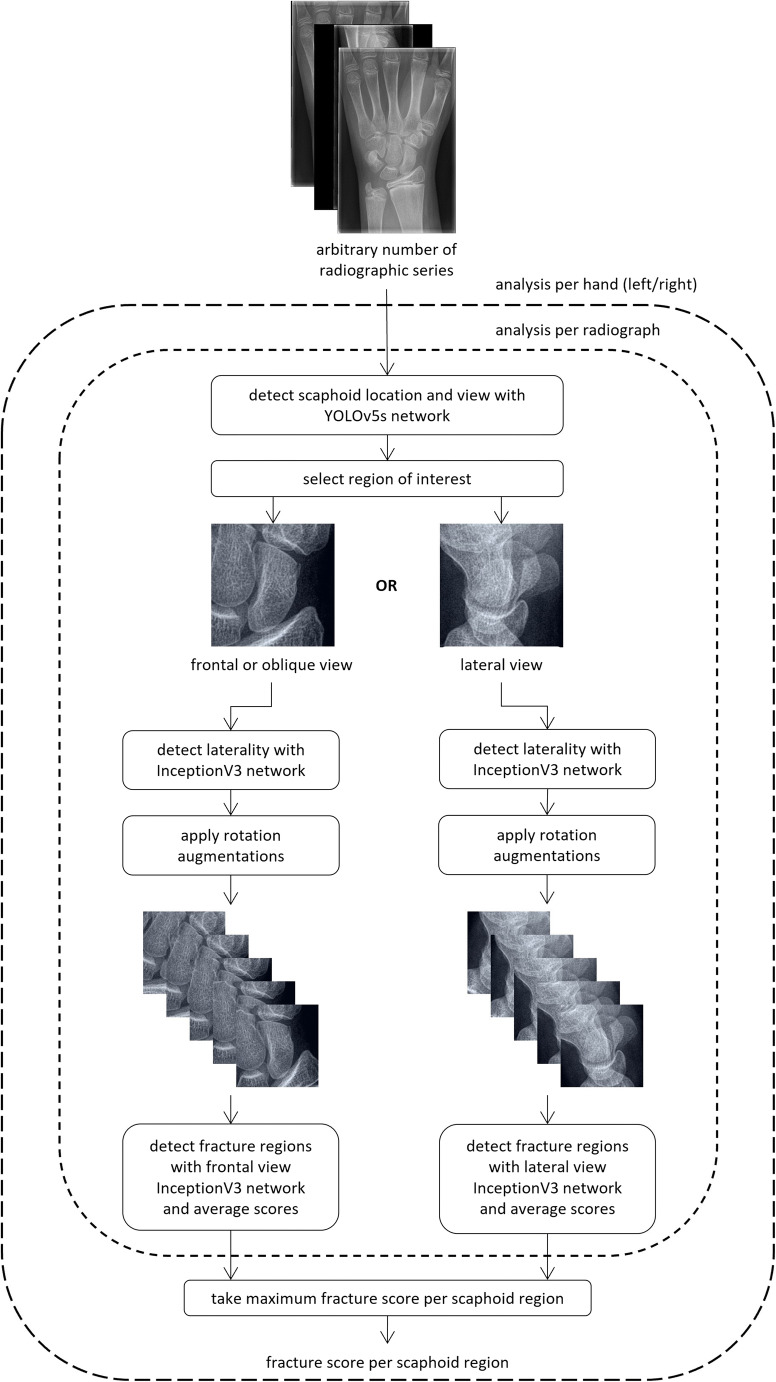
Overview of the scaphoid fracture detection artificial intelligence (AI) pipeline, which consisted of four convolutional neural networks (CNNs): a scaphoid localization CNN, scaphoid laterality classification CNN, and two scaphoid fracture detection CNNs for processing frontal or oblique view radiographs (including anterior-posterior/posterior-anterior [AP/PA] and ulnar-deviated AP/PA views) and lateral view radiographs separately

The pipeline of the scaphoid fracture detection AI algorithm is summarized in Fig. 2. The algorithm was designed for processing a radiographic study with an arbitrary number of series and it was implemented on an NVIDIA RTX Titan graphics processing unit with the PyTorch machine learning framework [19]. First, the scaphoid localization and laterality classification CNN localized the scaphoid and determined its orientation (frontal view, including [ulnar-deviated] AP/PA and oblique, or lateral view) and laterality (left or right hand). The scaphoid was then extracted from the image and was analyzed by either a frontal view or lateral view fracture detection CNN. In this analysis, a fracture score was generated for each of the six scaphoid regions as defined by Wong and Ho [18]. All processing steps were repeated for every input image and finally the maximum fracture score per region and per hand was selected. The whole algorithm is freely available at https://grand-challenge.org/algorithms/multiview-scaphoid-fracture-detection/, where it can be run in a web browser. A detailed description of the processing steps and training procedure is provided in Appendix E5 and E6 (online).

### Observer study

To validate the performance of the fracture detection AI algorithm as well as its potential value as a computer-aided diagnosis system, an observer study was conducted among five experienced MSK radiologists with 7, 5, 22, 24, and 26 years of experience (S.B., S.S., B.M., M.d.J., M.M.). For each patient in dataset 4, the radiologists independently assessed each of the six scaphoid regions as defined by Wong and Ho [18] for the presence of a fracture. They indicated their confidence for each region on a continuous scale from 0 to 1.0, where 1.0 indicates absolute certainty of a fracture and 0.5 is the cut-off point for determining whether a fracture was present. In cases where radiographs of both hands were taken, the radiologist indicated to which hand(s) their ratings applied. After a 4-month washout period, the radiologists repeated assessing all cases using the same protocol while being provided with the predictions of the algorithm. To minimize potential recall bias after the washout period, the order of the cases was shuffled.

### Statistical analysis

The auxiliary scaphoid localization and laterality classification CNN were separately evaluated on datasets 1 and 2. The evaluation details are provided in Appendix E7 (online). The fracture detection AI algorithm was cross-validated on dataset 3 using 10 folds (no patient overlap) and was tested on dataset 4. The evaluation metrics included the following: sensitivity, specificity, PPV, Cohen’s kappa coefficient (κ), area under the receiver operating characteristic curve (AUC), mean precision in localizing the fracture locations per scaphoid (“mean localization precision” [MLP]), and reading time (in seconds). The detection threshold that maximized the F1-score was chosen for the analysis. The fracture scores of the algorithm were based on automated image crops and laterality labels. The radiographic inputs from datasets 3 and 4 were respectively grouped by study and patient, and the corresponding scores were grouped by hand. Cohen’s κ was used for measuring the agreement between the algorithm and radiologists.

The evaluation metrics were calculated using the scikit-learn machine learning library (version 0.23.2, 2021) [20] for Python. Stratified bootstrapping with 1000 iterations was applied for estimating 95% confidence intervals (CIs). Significance testing was performed with two-sided paired permutation tests with 1000 iterations using the MLxtend library (version 0.19.0, 2021) [21] for Python. A difference with a *p* value smaller than .05 was considered significant.

## Results

### Test dataset characteristics

From the initial sample of 292 studies selected for the test dataset (dataset 4), 20 studies were excluded due to radiographs unsuitable for fracture diagnosis (*n* = 7), inconclusive evidence (*n* = 7), and non-acute fractures (*n* = 6) (see Fig. 1). This resulted into a final selection of 272 studies from 209 patients (mean age, 39 years ± 23 [standard deviation]; 107 women). The studies were grouped by patient and hand into 219 cases, of which 65 cases contained a scaphoid fracture (see Table 1). There was at least one PA view in all cases and there was at least one ulnar-deviated PA, oblique, and lateral view in 55 cases (30 with fracture), 159 cases (52 with fracture), and 156 cases (63 with fracture), respectively.

### Fracture detection by AI

**Fig. 3:**
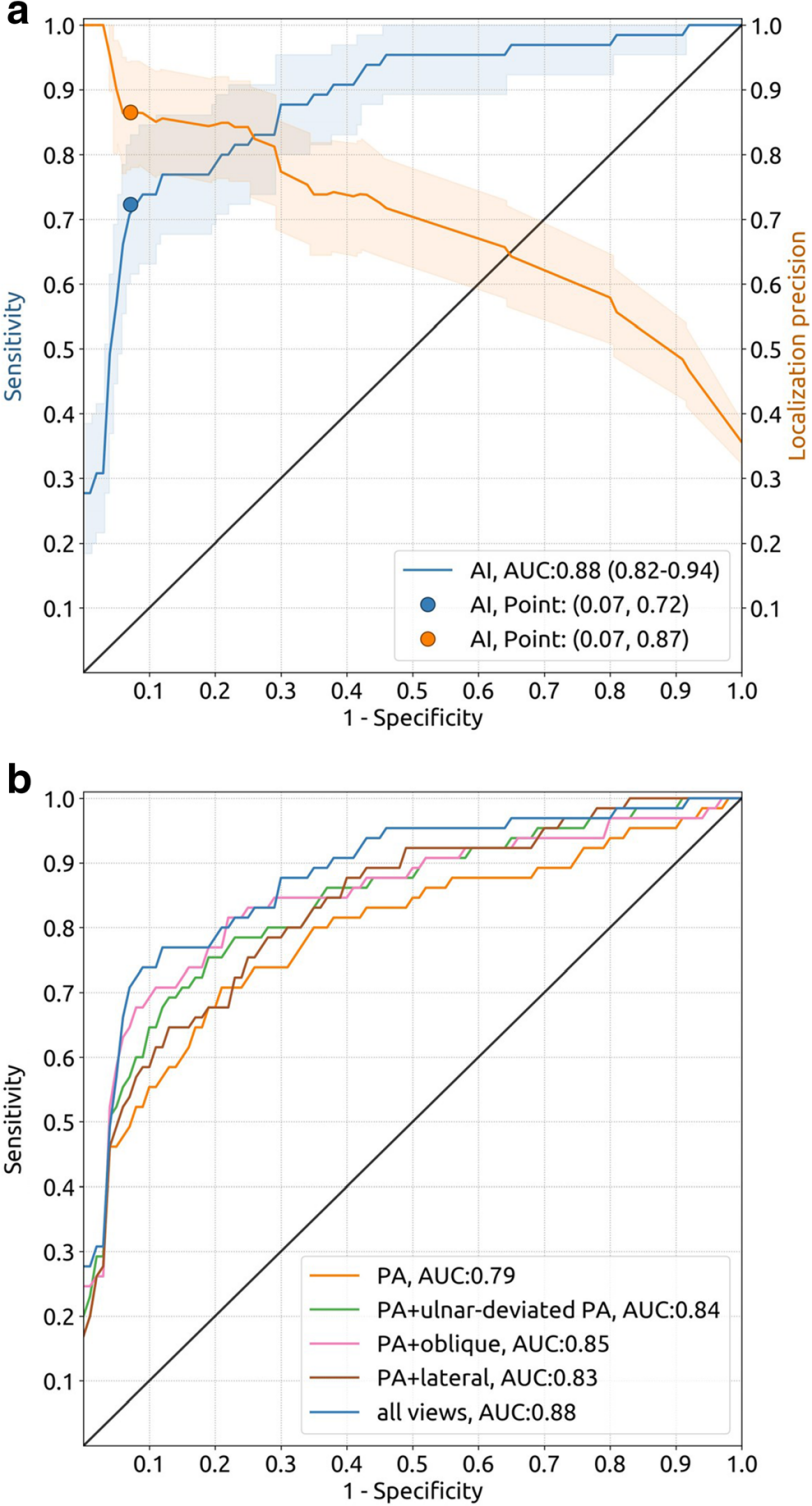
**a** Receiver operating characteristic (ROC) curve (blue) with operating point of the automated scaphoid fracture detection results based on all available radiographic views from dataset 4 (65 fracture cases, 154 non-fracture cases; each case represents one hand from one patient). The corresponding mean localization precision curve (orange) is shown as well. The shaded bands represent 95% confidence intervals. The black line represents no ability to discriminate between fracture and non-fracture cases. **b** Receiver operating characteristic (ROC) curves of the automated scaphoid fracture detection results for multiple input configurations on the same dataset. All 219 cases included at least one posterior-anterior (PA) view (65 cases with fracture), 55 cases included at least one ulnar-deviated PA view (30 cases with fracture), 159 cases included at least one oblique view (52 cases with fracture), and 156 cases included at least one lateral view (63 with fracture)

A quantitative and qualitative analysis of the scaphoid localization and laterality classification results are included in Appendix E8 (online). The scaphoid fracture detection AI algorithm obtained an AUC of 0.89 (95% CI: 0.87, 0.91) on dataset 3. The corresponding ROC curve and additional evaluation metrics are included in Appendix E9 (online). Table 2 presents the sensitivity, specificity, PPV, MLP, and AUC with their 95% CIs of the scaphoid fracture detection AI algorithm for multiple input configurations on the test dataset (dataset 4). The results are presented for all views and for each combination of PA views and one of the following views: ulnar-deviated PA, oblique, and lateral. The ROC curve with operation points and MLP curve (with 95% CI bands) for all views is shown in Fig. 3a. The ROC curve of each input configuration is shown in Fig. 3b. The fracture detection performance of the algorithm increased when PA views were supplemented with ulnar-deviated PA views (AUC, 0.79 vs. 0.84, *p* = .002), oblique views (AUC, 0.79 vs. 0.85, *p* = .02), and all available views (AUC, 0.79 vs. 0.88, *p* = .01), but not with lateral views (AUC, 0.79 vs. 0.83, *p* = .12). The median processing time per case (all inputs) and per radiograph was 0.97 and 0.28 s, respectively.

**Table 2:** Scaphoid fracture detection results of the AI

Input	Sensitivity (%)		Specificity (%)		PPV (%)		AUC		MLP (%)
	Value	Proportion	Value	Proportion	Value	Proportion	Value	*p*	Value
PA	51 (39, 63)	33/65	93 (89, 97)	143/154	75 (64, 86)	33/44	0.79 (0.71, 0.87)		86 (77, 95)
PA + ulnar-deviated PA	59 (46, 71)	38/65	93 (88, 97)	143/154	78 (67, 88)	38/49	0.84 (0.77, 0.91)	.002	88 (79, 96)
PA + oblique	66 (54, 77)	43/65	93 (89, 97)	143/154	80 (71, 89)	43/54	0.85 (0.77, 0.92)	.02	88 (79, 95)
PA + lateral	55 (42, 68)	36/65	93 (89, 97)	143/154	77 (65, 88)	36/47	0.83 (0.76, 0.90)	.12	83 (72, 93)
All views	72 (62, 83)	47/65	93 (88, 97)	143/154	81 (72, 91)	47/58	0.88 (0.82, 0.94)	.01	87 (78, 94)

Note. 95% CIs are shown in parentheses. The *p* value refers to the difference in AUC with the posterior-anterior (PA) view input configuration. The detection threshold was set to 0.616 for all input configurations. *AUC*, area under the receiver operating characteristic curve; *MLP*, mean localization precision; *PA*, posterior-anterior; *PPV*, positive predictive value

### Radiologist performance in scaphoid fracture detection with and without AI assistance

**Fig. 4:**
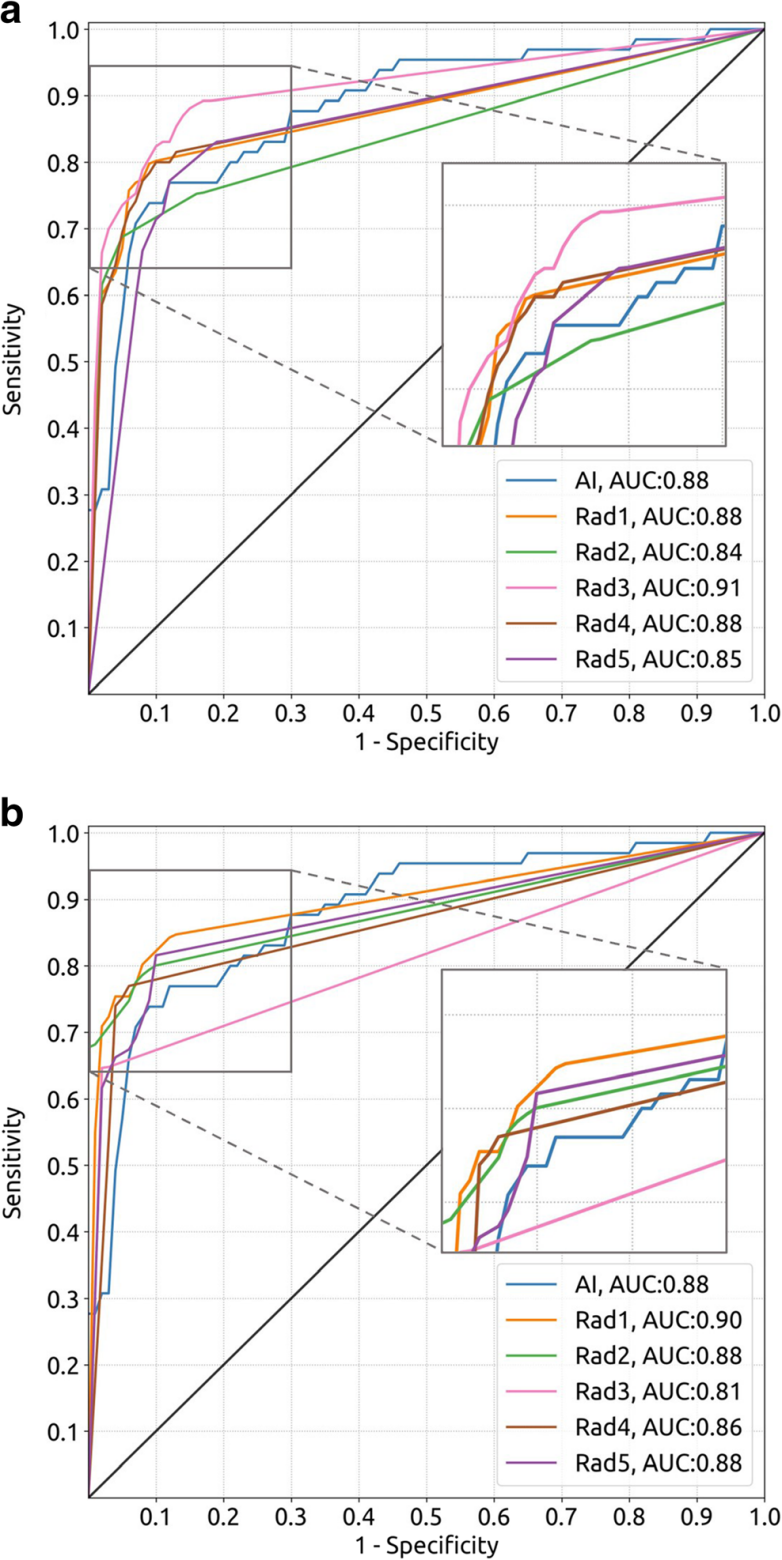
**a** Receiver operating characteristic (ROC) curves of the results of the scaphoid fracture detection algorithm and those of the musculoskeletal (MSK) radiologists without artificial intelligence (AI) assistance on dataset 4 (65 fracture cases, 154 non-fracture cases; each case represents one hand from one patient). **b** ROC curves of the results of the scaphoid fracture detection algorithm and those of the MSK radiologists with AI assistance on the same dataset. The corner of each plot is magnified for easier comparison of the curves. The black line represents no ability to discriminate between fracture and non-fracture cases.

Table 3 presents the sensitivity, specificity, PPV, MLP, AUC, and median reading time with their 95% CIs and *p* values of the five MSK radiologists for scaphoid fracture detection with and without AI assistance. The corresponding ROC curves are shown in Fig. 4a and b. The ROC curves with operating points and MLP per radiologist are shown in Appendix E10 (online). With AI assistance, three radiologists obtained a higher specificity (Rad2, 94% vs. 84%, *p* = < .001; Rad3, 97% vs. 88%, *p* = .003; Rad5, 90% vs. 81%, *p* = .03), three radiologist obtained a higher PPV (Rad2, 83% vs. 66%, *p* = < .001; Rad3, 91% vs. 74%, *p* = .006; Rad5, 77% vs. 65%, *p* = .04), one radiologist obtained a lower AUC (Rad3, 0.81 vs. 0.91, *p* = .002), and four radiologists had a lower reading time (Rad2, 27 vs. 16, *p* = < .001, Rad3, 21 vs. 11, *p* = < .001, Rad4, 13 vs. 6, *p* = < .001, Rad5, 35 vs. 14, *p* = < .001). In all other cases, AI assistance had no effect on the sensitivity, specificity, PPV, MLP, AUC, and reading time.

**Table 3:** Scaphoid fracture detection results of the radiologists

Condition	Reader	Sensitivity (%)			Specificity (%)			PPV (%)		
		Value	Proportion	*p*	Value	Proportion	*p*	Value	Proportion	*p*
Without AI assistance	Rad1	75 (65, 86)	49/65		94 (90, 97)	145/154		85 (77, 93)	49/58	
	Rad2	75 (66, 86)	49/65		84 (78, 90)	129/154		66 (58, 75)	49/74	
	Rad3	83 (74, 91)	54/65		88 (82, 93)	135/154		74 (66, 83)	54/73	
	Rad4	80 (71, 89)	52/65		91 (86, 96)	140/154		79 (70, 88)	52/66	
	Rad5	83 (74, 91)	54/65		81 (75, 87)	125/154		65 (58, 73)	54/83	
With AI assistance	Rad1	72 (61, 83)	47/65	.77	97 (94, 99)	149/154	.32	90 (83, 98)	47/52	.28
	Rad2	75 (65, 85)	49/65	> .99	94 (90, 97)	144/154	< .001	83 (75, 92)	49/59	< .001
	Rad3	65 (52, 77)	42/65	.007	97 (95, 99)	150/154	.003	91 (84, 98)	42/46	.006
	Rad4	77 (66, 86)	50/65	.76	94 (90, 97)	145/154	.34	85 (77, 93)	50/59	.30
	Rad5	77 (66, 86)	50/65	.42	90 (85, 95)	139/154	.03	77 (68, 86)	50/65	.04
Condition	Reader	AUC			MLP (%)			Median reading time (s)		
		Value		*p*	Value		*p*	Value	*p*	
Without AI assistance	Rad1	0.88 (0.82, 0.93)			94 (87, 99)			22 (18, 26)		
	Rad2	0.84 (0.78, 0.90)			97 (93, 100)			27 (24, 29)		
	Rad3	0.91 (0.86, 0.96)			91 (82, 98)			21 (19, 26)		
	Rad4	0.88 (0.82, 0.93)			91 (84, 97)			13 (12, 16)		
	Rad5	0.85 (0.80, 0.90)			94 (89, 99)			35 (29, 44)		
With AI assistance	Rad1	0.90 (0.84, 0.95)		.18	98 (95, 100)		.33	30 (24, 40)	.06	
	Rad2	0.88 (0.83, 0.93)		.14	97 (93, 100)		> .99	16 (13, 19)	< .001	
	Rad3	0.81 (0.75, 0.87)		.002	95 (88, 100)		.34	11 (9, 12)	< .001	
	Rad4	0.86 (0.81, 0.91)		.57	91 (82, 98)		.95	6 (5, 7)	< .001	
	Rad5	0.88 (0.83, 0.93)		.39	100 (100, 100)		.10	14 (12, 16)	< .001	

Note. 95% CIs are shown in parentheses. The *p* values refer to the differences in evaluation metrics between the with and without AI assistance condition. *AUC*, area under the receiver operating characteristic curve; *MLP*, mean localization precision; *PPV*, positive predictive value; *s*, seconds

Table 4 shows the Cohen’s kappa coefficients with their 95% CIs and *p* values for the fracture detection agreements between the radiologists (with and without AI assistance) and the AI algorithm. Overall, the radiologists were in a moderate to substantial agreement with each other (range without AI assistance: 0.50–0.71; range with AI assistance: 0.62–0.79). With AI assistance, five out of the ten pairs of radiologists had a higher agreement (Rad1-Rad3, 0.56 vs. 0.79, *p* = .002; Rad2-Rad3, 0.50 vs. 0.71, *p* = .006; Rad2-Rad4, 0.58 vs. 0.74, *p* = .02; Rad3-Rad4, 0.53 vs. 0.74, *p* = .003; Rad3-Rad5, 0.52 vs. 0.68, *p* = .03), whereas the agreement between all other pairs of radiologists remained unchanged. With AI assistance, two out of the five radiologists had a higher agreement with the algorithm (Rad3, 0.56 vs. 0.72, *p* = .02; Rad4, 0.62 vs. 0.80, *p* = < .001), whereas all other radiologists agreed equally well with the algorithm. The proportion of correctly and incorrectly changed fracture diagnoses by the radiologists with AI assistance and their correlation with the automated fracture scores are shown in Appendix E11 (online).

**Table 4:** Scaphoid fracture detection agreement between radiologists and AI in terms of Cohen’s kappa

Condition		Kappa coefficients reader pairs									
		Rad1		Rad2		Rad3		Rad4		Rad5	
		Value	*p*	Value	*p*	Value	*p*	Value	*p*	Value	*p*
Without AI assistance	AI	0.65 (0.54, 0.75)		0.57 (0.46, 0.68)		0.56 (0.43, 0.67)		0.62 (0.51, 0.73)		0.56 (0.44, 0.67)	
	Rad1			0.59 (0.48, 0.71)		0.56 (0.43, 0.68)		0.71 (0.61, 0.81)		0.56 (0.44, 0.66)	
	Rad2					0.50 (0.37, 0.61)		0.58 (0.47, 0.68)		0.57 (0.46, 0.68)	
	Rad3							0.53 (0.40, 0.64)		0.52 (0.40, 0.64)	
	Rad4									0.59 (0.47, 0.69)	
With AI assistance	AI	0.66 (0.54, 0.77)	.84	0.64 (0.52, 0.75)	.23	0.72 (0.61, 0.83)	.02	0.80 (0.71, 0.89)	< .001	0.60 (0.49, 0.72)	.44
	Rad1			0.72 (0.61, 0.82)	.05	0.79 (0.69, 0.88)	.002	0.70 (0.59, 0.81)	.91	0.62 (0.50, 0.73)	.43
	Rad2					0.71 (0.60, 0.81)	.006	0.74 (0.64, 0.83)	.02	0.64 (0.52, 0.75)	.36
	Rad3							0.74 (0.63, 0.83)	.003	0.68 (0.57, 0.78)	.03
	Rad4									0.69 (0.57, 0.78)	.19

Note. 95% CIs are shown in parentheses. The test dataset (dataset 4) consisted of 65 fracture cases and 154 non-fracture cases

### Comparison of AI performance with experienced MSK radiologists

The AI algorithm and the MSK radiologists (unassisted) had a similar performance in detecting scaphoid fractures (AUC, 0.88 vs. 0.87 [average of radiologists, range: 0.84–0.91], see Tables 2 and 3; Rad1, *p* = .89; Rad2, *p* = .32; Rad3, *p* = .32, Rad4, *p* = .90; Rad5, *p* = .46). Two radiologists had a higher MLP than the algorithm (Rad2, 97% vs. 87%, *p* = .01; Rad5, 94% vs. 87%, *p* = .048), whereas there was no difference in MLP between the other radiologists and the algorithm (92% [average of radiologists, range: 91–94%] vs. 87%, see Tables 2 and 3; Rad1, *p* = .11, Rad3, *p* = .21, Rad4, *p* = .24).

**Fig. 5:**
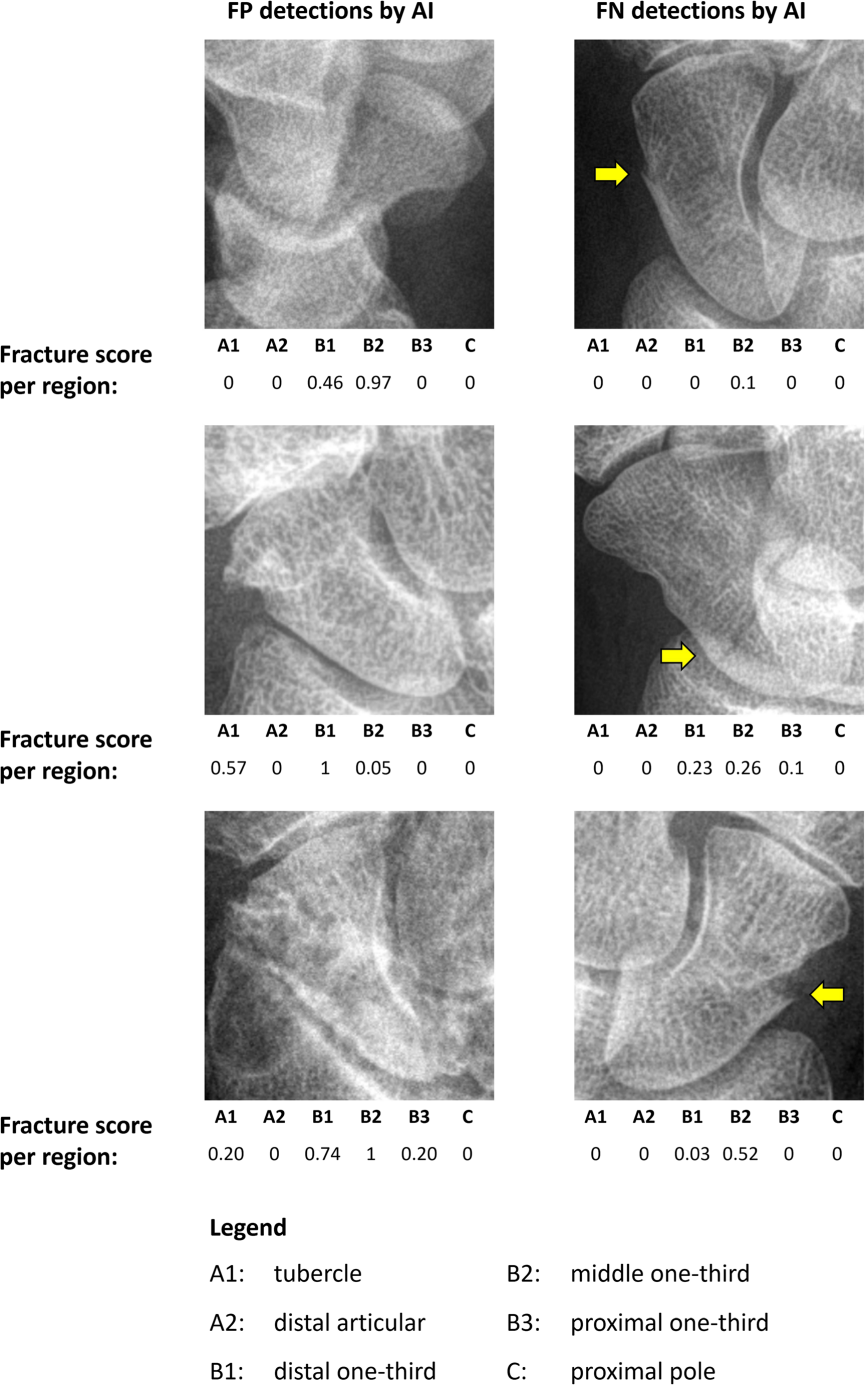
False positive (FP) and false negative (FN) detections made by the scaphoid fracture detection artificial intelligence (AI) algorithm that none of the five musculoskeletal radiologists made. The AI fracture score per scaphoid region (ranging from 0 [no fracture] to 1 [fracture], rounded to two decimals) is shown below each image. The yellow arrows indicate the fracture locations and are only shown for reference. False positive case descriptions (from top to bottom): 13-year-old male and 77-year-old female with an intact scaphoid, 81-year-old male with rheumatoid arthritis with an old healed waist scaphoid fracture. False negative case descriptions (from top to bottom): 67-year-old female with a slightly displaced transverse waist scaphoid fracture (transition middle one-third to distal one-third), 37-year-old male with a waist oblique scaphoid fracture (transition proximal one-third to middle one-third), 77-year-old female with a waist scaphoid fracture (middle one-third)

**Fig. 6:**
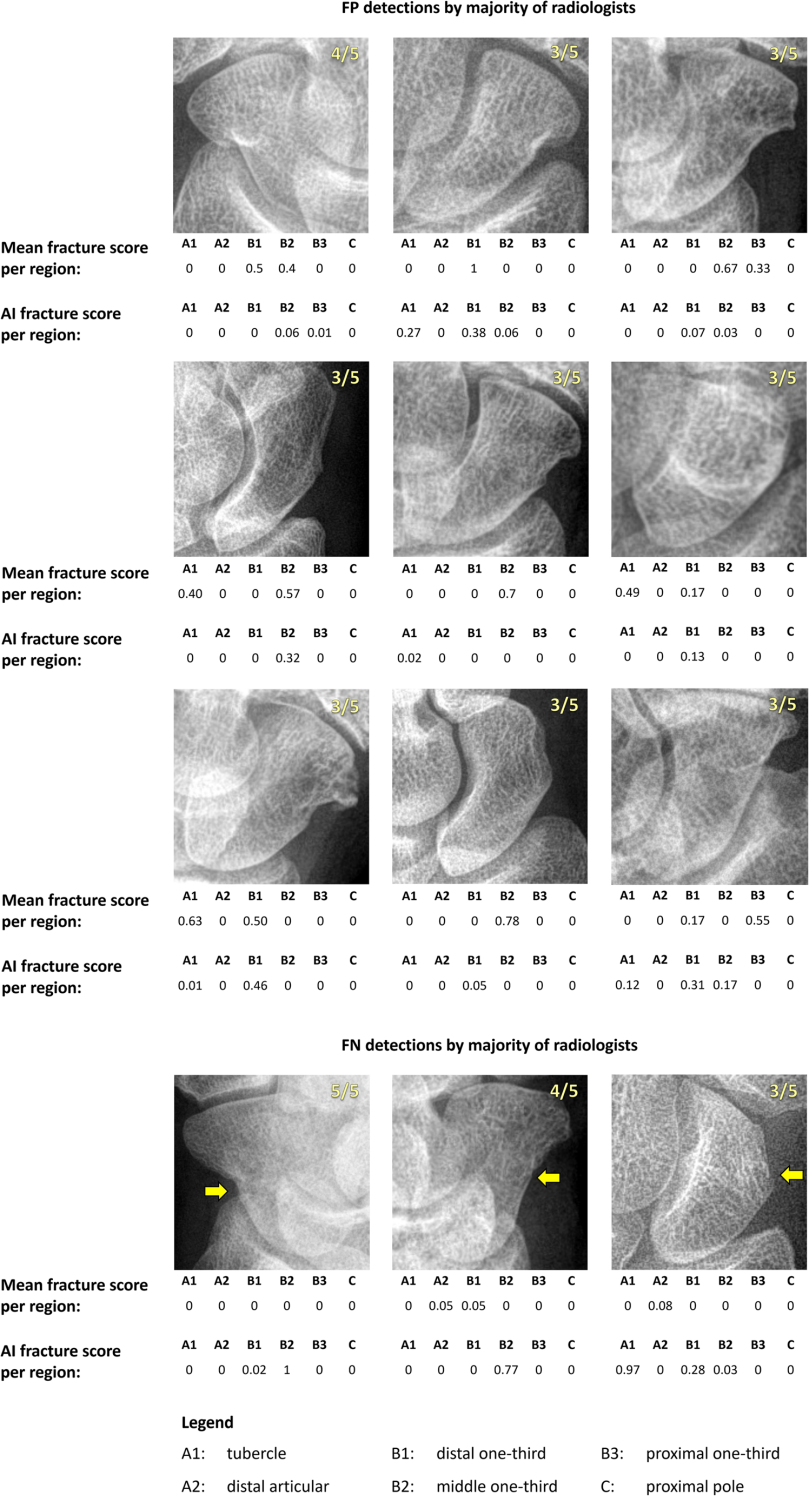
False positive (FP) and false negative (FN) detections made by the majority of the five musculoskeletal radiologists that the artificial intelligence (AI) algorithm did not make. The proportion of radiologists making the FP or FN detection is shown in the upper right corner of each image. The corresponding fracture scores per scaphoid region of the radiologists (mean score of responsible radiologists per region) and AI (ranging from 0 [no fracture] to 1 [fracture], rounded to two decimals) are shown below each image. The yellow arrows indicate the fracture locations and are only shown for reference. Case descriptions first row (left to right): 27-year-old female, 12-year-old male, and 50-year-old female with an intact scaphoid. Case descriptions second row (left to right): 74-year-old female and 59-year-old female with an intact scaphoid, 79-year-old female with calcium pyrophosphate deposition arthritis with calcifications surrounding the triangular fibrocartilage complex. Case descriptions third row (left to right): 69-year-old female with osteophyte and subchondral cyst formation, 45-year-old female with an intact scaphoid, 74-year-old male with radiocarpal and scapho-trapezium/trapezoid joint arthritis. Case descriptions fourth row (left to right): 23-year-old male with a waist scaphoid fracture (middle one-third), 60-year-old female with a waist scaphoid fracture (distal one-third), 12-year-old female with a waist scaphoid fracture (middle one-third)

A follow-up analysis of the decisions made by the algorithm and MSK radiologists revealed that six out of the 29 mistakes of the algorithm (three fracture cases, three non-fracture cases) were not made by any of the radiologists. Conversely, 12 out of the 23 mistakes made by the majority of radiologists (three fracture cases, nine non-fracture cases) were not made by the algorithm. The failure cases of the algorithm and radiologists are shown in Figs. 5 and 6 respectively.

## Discussion

Patients with a clinically suspected scaphoid fracture often receive unnecessary wrist immobilization, as acute scaphoid fractures can cause severe damage to the wrist if they remain undetected and untreated. This study assessed how a CNN-based AI algorithm performed against five MSK radiologists and whether it aided diagnosis of scaphoid fractures on conventional multi-view radiographs. The algorithm was shown to be able to detect scaphoid fractures as well as the MSK radiologists (AUC, 0.88 vs. 0.87 [average of radiologists]; *p* ≥ .05 for all radiologists). Moreover, it was able to indicate which regions in the scaphoid were fractured as precisely as the majority of radiologists (MLP, 87% vs. 92% [average of radiologists], *p* ≥ .05 for majority). Furthermore, AI assistance improved five out of ten pairs of inter-observer Cohen’s κ agreement (average increase of 36.2%, *p* < .05 for all pairs) and reduced the reading time of four radiologists (average reduction of 49.4%, *p* < .001 for all radiologists), but no improvements were found in sensitivity, specificity, PPV, and AUC for the majority of radiologists.

The results showed that the scaphoid fracture detection performance of the AI algorithm improved when PA views were supplemented with ulnar-deviated and oblique PA views, whereas adding lateral views did not lead to a performance increase. These findings underline the conclusions of Cheung et al [22] that the PA, ulnar-deviated PA and oblique view are most important for scaphoid fracture detection and indicate that this also applies to deep learning–based AI algorithms. This implies that a multi-view approach to scaphoid fracture detection should be adapted in future AI applications.

A qualitative analysis of the failure cases revealed that the algorithm made six mistakes (three fracture cases, three non-fracture cases) that none of the MSK radiologists made. The false positive detections were likely to be caused by overprojection lines of the other carpal bones on the scaphoid on the lateral view and a sclerotic line from an old healed fracture. The false negative cases included two scaphoids with an evident, but non-sharply delineated scaphoid waist fracture. The latter finding is in line with the observations of Hendrix et al [12] and Langerhuizen et al [23] that deep learning–based AI algorithms may miss fractures that are evident to human observers.

There were 12 mistakes made by the majority of MSK radiologists (three fracture cases, nine non-fracture cases) that were not made by the algorithm. In most of the false positive cases (5/9), the scaphoid and its surrounding joints showed degenerative signs or slight deformities, which could suggest a fracture even when no hypodense line was visible. The remaining false positive detections were caused by very subtle or diffuse hypodense lines. The false negative detections were made in scaphoids with a displaced fracture causing a subtle protrusion of the cortical bone with no evident fracture line visible. Similar false negative detections made by radiologists but not by an AI algorithm can be observed in Hendrix et al [12], but qualitative analyses of false positive detections are lacking in previous studies. While these findings suggest that the algorithm may have merit in aiding the interpretation of degenerative or malformed scaphoids for fracture diagnosis, follow-up studies are required to confirm this.

The found positive effects of AI assistance on the inter-observer agreement and reading time of the radiologists provide preliminary evidence that the algorithm could improve the diagnostic efficiency of MSK radiologists with the same diagnostic accuracy. However, one radiologist had a significantly lower sensitivity and AUC in the AI assistance condition, but the incorrectly changed answers were weakly correlated with the answers from the algorithm (0.34). The decreases in reading time are in line with the conclusions from Duron et al [14] and Guermazi et al [15], but we did not find any increases in sensitivity or consistent increases in specificity. This difference could be due to the few carpal fractures in their test datasets. Furthermore, even though it was not investigated in this study, it could be expected that general radiologists, radiology residents, and other physicians may benefit more from AI assistance.

The strengths of this study included the use of multi-center clinical data and external validation, participation of five experienced MSK radiologists, and the evaluation of an automatic multi-view scaphoid fracture detection AI algorithm. However, this study also had some limitations. First, we aimed to minimize selection bias in our test set by using all available information in the PACS and EHR system rather than using only studies with a follow-up CT or MRI scan for testing. This means that occult scaphoid fractures might have been labelled as negative cases when the patient did not return to the hospital with persistent symptoms. The reference standard quality and selection bias trade-off problem could be circumvented by conducting a prospective study in which patients immediately undergo a CT and MRI scan after an initial examination with conventional radiography, but this would be too time intensive and costly for acquiring sufficient data.

Second, even though we investigated the contribution of each radiographic view to automated scaphoid fracture detection, we simplified the model architecture by processing AP/PA, ulnar-deviated AP/PA, and oblique view radiographs by a single CNN. The model performance might be further improved in future research by training separate CNNs for all views.

In conclusion, the findings presented in this study support the hypothesis that an AI algorithm can achieve MSK radiologist level performance in detecting scaphoid fractures on conventional multi-view radiographs. Moreover, there is preliminary evidence that AI assistance could improve the diagnostic efficiency of MSK radiologists, but not their diagnostic accuracy. Future research should evaluate the impact of AI assistance on diagnostic performance, clinical decision making, and patient outcomes in a randomized clinical trial involving both radiologists and non-radiologists.

## Supplementary Information

ESM 1 (DOCX 1.16 mb)

## Abbreviations

AUC: Area under the receiver operating characteristic curve
CNN: Convolutional neural network
MLP: Mean localization precision
MSK: Musculoskeletal
PPV: Positive predictive value

## Declarations

### Guarantor

The scientific guarantor of this publication is Nils Hendrix.

### Statistics and biometry

One of the authors has significant statistical expertise.

### Study subjects or cohorts overlap

Some study subjects or cohorts have been previously reported in the following publication: *Hendrix N, Scholten E, Vernhout B, et al (2021) Development and Validation of a Convolutional Neural Network for Automated Detection of Scaphoid Fractures on Conventional Radiographs. Radiol Artif Intell 3(4):e200260.*

### Methodology

retrospective

experimental

multicenter study

## References

[1] Rhemrev SJ, Ootes D, Beeres FJP, Meylaerts SAG, Schipper IB (2011). Current methods of diagnosis and treatment of scaphoid fractures. Int J Emerg Med.

[2] de Zwart AD, Beeres FJP, Rhemrev SJ, Bartlema K, Schipper IB (2016). Comparison of MRI, CT and bone scintigraphy for suspected scaphoid fractures. Eur J Trauma Emerg Surg.

[3] Tiel-van Buul MM, van Beek EJ, Broekhuizen AH, Bakker AJ, Bos KE, van Royen EA (1993). Radiography and scintigraphy of suspected scaphoid fracture. A long-term study in 160 patients. J Bone Joint Surg Br.

[4] Gibney B, Smith M, Moughty A, Kavanagh EC, Hynes D, MacMahon PJ (2019). Incorporating cone-beam CT into the diagnostic algorithm for suspected radiocarpal fractures: a new standard of care?. AJR Am J Roentgenol.

[5] Grewal R, Lutz K, MacDermid JC, Suh N (2016). Proximal pole scaphoid fractures: a computed tomographic assessment of outcomes. J Hand Surg Am.

[6] Clementson M, Björkman A, Thomsen NOB (2020). Acute scaphoid fractures: guidelines for diagnosis and treatment. EFORT Open Rev.

[7] Burns MJ, Aitken SA, McRae D, Duckworth AD, Gray A (2013). The suspected scaphoid injury: resource implications in the absence of magnetic resonance imaging. Scott Med J.

[8] Blum A, Sauer B, Detreille R (2007). The diagnosis of recent scaphoid fractures: review of the literature. J Radiol.

[9] Karl JW, Swart E, Strauch RJ (2015). Diagnosis of occult scaphoid fractures: a cost-effectiveness analysis. J Bone Joint Surg Am.

[10] Yin ZG, Zhang JB, Gong KT (2015). Cost-effectiveness of diagnostic strategies for suspected scaphoid fractures. J Orthop Trauma.

[11] Kuo RYL, Harrison C, Curran TA et al (2022) Artificial intelligence in fracture detection: a systematic review and meta-analysis. Radiology. 10.1148/radiol.211785 10.1148/radiol.211785 PMC9270679 35348381

[12] Hendrix N, Scholten E, Vernhout B (2021). Development and validation of a convolutional neural network for automated detection of scaphoid fractures on conventional radiographs. Radiol Artif Intell.

[13] Yoon AP, Lee YL, Kane RL, Kuo CF, Lin C, Chung KC (2021). Development and validation of a deep learning model using convolutional neural networks to identify scaphoid fractures in radiographs. JAMA Netw Open.

[14] Duron L, Ducarouge A, Gillibert A (2021). Assessment of an AI aid in detection of adult appendicular skeletal fractures by emergency physicians and radiologists: a multicenter cross-sectional diagnostic study. Radiology.

[15] Guermazi A, Tannoury C, Kompel AJ (2022). Improving radiographic fracture recognition performance and efficiency using artificial intelligence. Radiology.

[16] Yang TH, Horng MH, Li RS, Sun YN (2022). Scaphoid fracture detection by using convolutional neural network. Diagnostics (Basel).

[17] Tung YC, Su JH, Liao YW (2021). High-performance scaphoid fracture recognition via effectiveness assessment of artificial neural networks. Appl Sci.

[18] Wong WYC, Ho PC (2011). Minimal invasive management of scaphoid fractures: from fresh to nonunion. Hand Clin.

[19] Paszke A, Gross S, Massa F (2019). PyTorch: an imperative style, high-performance deep learning library. Adv Neural Inf Proces Syst.

[20] Pedregosa F, Varoquaux G, Gramfort A (2011). Scikit-learn: machine Learning in Python. J Mach Learn Res.

[21] Raschka S (2018). MLxtend: providing machine learning and data science utilities and extensions to Python’s scientific computing stack. J Open Source Softw.

[22] Cheung GC, Lever CJ, Morris AD (2006). X-ray diagnosis of acute scaphoid fractures. J Hand Surg Br.

[23] Langerhuizen DWG, Bulstra AEJ, Janssen SJ (2020). Is deep learning on par with human observers for detection of radiographically visible and occult fractures of the scaphoid?. Clin Orthop Relat Res.

