# Supplementary material for: Musculoskeletal radiologist-level performance by using deep learning for detection of scaphoid fractures on conventional multi-view radiographs of hand and wrist
Source: Eur Radiol. 2022 Nov 15;33(3):1575–88. doi: 10.1007/s00330-022-09205-4 (PMC9935716; doi:10.1007/s00330-022-09205-4)

**Online supplement**

**Appendix E1.** Additional Imaging Parameters for Table 1

Table E1 shows an overview of additional imaging parameters for the radiographs in the experimental datasets to complement the parameters provided in Table 1 in the main text.

**Appendix E2.** Annotation Protocol

The datasets were annotated by N.H. and an MSK radiologist with 26 years of experience (M.R.). For all datasets, scaphoid bounding boxes (except dataset 4), radiographic view labels (AP/PA, ulnar-deviated AP/PA, oblique, lateral), and laterality labels (scaphoid from left/right hand) were created with the software Visual Geometry Group (VGG) Image Annotator (1). The AP/PA, ulnar-deviated AP/PA, and oblique views were aggregated to a single frontal view category when developing the AI algorithm, as these views are visually similar and the ulnar-deviated AP/PA formed a minority group.

For datasets 3 and 4, additional scaphoid fracture labels were created that indicated a fracture in six scaphoid regions, as defined by Wong and Ho (2). These regions included: scaphoid tubercle (A1), distal articular (A2), distal one-third (B1), middle one-third (B2), proximal one-third (B3), and proximal pole (C). If a given fracture line crossed multiple regions, all underlying regions were marked as being fractured. For dataset 3, the original radiology reports were consulted to determine the fracture presence. When a report was inconclusive, the corresponding study was re-evaluated by radiologist M.R.

**Appendix E3.** Inclusion and Exclusion Criteria

Figures E1 and E2 show the flowcharts for the inclusion and exclusion of patients and radiographic studies in respectively the training data (datasets 1 and 3) and test data (dataset 2, see flowchart for dataset 4 in the main text). The sample sizes were largely determined by the availability of suitable radiographic studies and in particular by the available number of scaphoid fracture studies (dataset 3). It is important to note that the scaphoid segmentation dataset from Hendrix et al (3) was extended and used for training a scaphoid detector for a first pass annotation of additional data in dataset 1, as shown in Figure E1. The following main inclusion and exclusion criteria were used for selecting the radiographic studies:

1. Exclude studies when the scaphoid is occluded by a casts or metal osteosynthesis material. The occlusion of the scaphoid impedes the fracture diagnosis and may bias the deep learning model. Studies with wrists in cast were included in datasets 1 and 2 when the overprojection on the scaphoid was limited and accurate scaphoid localization was still possible.
2. Exclude studies when the scaphoid is resected, not grown (infants), or unrecognizably malformed due to a pathology. The scaphoid cannot be detected under these circumstances.
3. Exclude studies depicting non-acute scaphoid fractures (i.e., delayed or non-union scaphoid fractures older than four weeks). This criterion was only used for dataset 4. Non-acute fractures are ill-defined and are dilated fractures which are easy to diagnose. Furthermore, the focus of the current study was on early scaphoid fracture detection. Past fractures that were fully consolidated were considered non-fractures.
4. Include only studies with a follow-up radiographic study between one and four weeks. This criterion was only used for selecting negative cases for dataset 3. A minimum and maximum period of respectively one and four weeks was used, because acute scaphoid fractures can be poorly visible before this period and subtle fractures may already be healed or no longer visible after this period.
5. Exclude studies with severe scapholunate advanced collapse where the proximal pole of the scaphoid cannot be adequately assessed for the presence of a fracture. This criterion was used for datasets 3 and 4.

**Appendix E4.** Reference Standards for Dataset 4.

Table E2 shows an overview of the available reference standards for patients with and without scaphoid fracture in dataset 4. The reference standards were divided into the following categories based on quality (from high to low): follow-up CT and MRI, follow-up MRI, follow-up CT, follow-up scaphoid radiographs, follow-up hand and wrist radiographs, previous imaging studies (conventional radiography, CT, and MRI of the hand and wrist), and clinical information only (including patient demographics, clinical history, and indication). Studies conducted within four weeks after the initial study were considered follow-up studies. The categories were mutually exclusive and patients were allocated to each category based on the highest available reference standard.

**Appendix E5.** Image Processing Steps

The following steps were taken for processing a given image in the AI pipeline:

1. *Convert image*
   1. Rescale the pixel data to a 16-bit intensity range. At training time, the Digital Imaging and Communications in Medicine (DICOM) files are converted to 16-bit Portable Network Graphics (PNG) in advance.
   2. Transform the image colour from gray-scale to RGB.
2. *Standardize image*
   1. Normalize the pixel spacing of the image using the Pixel (Imager) Spacing attribute from the original DICOM file. Use bilinear interpolation for image scaling. Save a copy of the original image.
   2. Fix the image size to 1600 × 1600 pixels by zero padding or center-cropping.
   3. Normalize the pixel values by first rescaling the values between 0 and 1 via min-max scaling and then zero centering the values using the per-channel mean and standard deviation of the ImageNet dataset (4) (mean: [0.485, 0.456, 0.406]; standard deviation: [0.229, 0.224, 0.225]). At training time, this step optimizes the transfer learning effect from the network initialization with weights pretrained on the ImageNet dataset.
3. *Remove irrelevant regions*
   1. Localize the scaphoid in the image with the scaphoid localization CNN and map the bounding box coordinates back to the original image dimensions. A lightweight YOLOv5s architecture (5) was chosen for this task, as it allowed for a high input resolution that preserves the visibility of the back of the scaphoid. It also classifies whether the scaphoid is depicted from the front or the side.
   2. Crop to the original image (saved at step 2a) to a 33 × 33 mm patch enclosing the scaphoid (use center coordinate of the bounding box). This way, limited context information can be used for the scaphoid laterality classification and fracture detection task, while reducing computational costs and potential overfitting during training. The patch size was determined based on the 99^th^ percentile of the scaphoid height and width in dataset 1. The image is cropped to a fixed-size patch instead of the original bounding box, so that any bounding box inaccuracies would not result in changes in scale at step 4b. Any rotation augmentation is applied before the cropping operation.
4. *Standardize image crop*
   1. Normalize the image crop contrast using the contrast stretching method. Use the minimum and maximum value in the bounding box in the original image as output value range.
   2. Resize the image crop to 299 × 299 pixels using bilinear interpolation. The chosen InceptionV3 network (6) for the laterality classification and fracture detection task (see next step) was designed for and pretrained with this pixel resolution.
   3. Repeat step 2c to normalize the pixel values.
5. *Detect scaphoid fractures*
   1. Detect and localize scaphoid fractures by processing the image crop with either the frontal or lateral fracture detector, depending on the predicted view at step 3a. The detector returns a fracture score from 0 (not fractured) to 1 (fractured) for each of the six scaphoid regions as defined by Wong and Ho (2). The InceptionV3 architecture (6) was chosen this task, because it is optimized for extracting multi-scale image features that could account for the different scales on which fractures can alter the scaphoid appearance.
   2. Apply a Test Time Augmentation (TTA) technique where the fracture analysis is repeated under four additional angles of rotation (10 and 20 degrees, both directions) to further improve the fracture detection performance. The corresponding fracture scores are averaged.
   3. Determine whether the scaphoid belongs to a right or left hand by processing the (non-augmented) image crop with the laterality classification CNN. An InceptionV3 architecture (6) was also chosen for this task. The laterality is visually determined, as this information may be unavailable in the metadata.
   4. Repeat all processing steps for each radiograph to be analysed and return the maximum fracture score per scaphoid region per hand.

**Appendix E6.** Training Procedure

A YOLOv5s network (version v5.0-196-gbb79e13) (5) was initialized with weights pretrained on the COCO dataset (7) and was finetuned on dataset 1. The training settings of the YOLOv5 framework were left at their default values, except for the batch size that was set to 4 and the colour augmentations (hue and saturation) that were disabled (more about data augmentation later on). At default, the weights of the YOLOv5 network were updated with the SGD optimizer with Nesterov momentum (learning rate = 0.01, momentum = 0.937, weight decay = 0.0005) (8) with learning rate and momentum warm-up. The network was trained for 550 epochs.

The scaphoid laterality classification CNN was initialized with weights pretrained on the ImageNet dataset (4) and was finetuned on dataset 1. The ADAM optimizer (9) (β1 = 0.9, β2 = 0.999) was used for weight optimization and minimized the categorical cross-entropy loss averaged over a mini-batch of eight images:

$$\frac{1}{N}\sum_{i=1}^{N} \sum_{j=1}^{C} -y_{i,j}\log\left( p_{i,j} \right)$$

where *N* is the number of images in the mini-batch, *C* is the number of classes, and *y_i,j_* and *p_i,j_* are respectively a binary label and probability indicating whether the image depicts a scaphoid from the left or right hand. Stratified mini-batch sampling was applied so that each mini-batch contained an equal number of images per class. The initial learning rate was set to 1×10^-4^ and it was reduced by a factor 10 whenever the loss did not decrease for 10 epochs (the minimum learning rate was 1×10^-6^). The training process was ended upon convergence.

The scaphoid fracture detection CNNs were trained on dataset 3 with the same protocol as for the scaphoid laterality classification CNN, with exception of the loss function. Since the fracture detection task was formulated as a multi-label classification problem with six classes (not mutually exclusive), the cross-entropy loss was calculated with sigmoid outputs instead of softmax outputs. Furthermore, a loss weighting scheme was applied where large localization mistakes were weighted more heavily than small localization mistakes: the loss multiplier was set to the number of steps the predicted fracture regions were removed from the true positive regions plus one. In case of multiple fracture regions, the minimum weighted loss per scaphoid region was selected. The loss of the auxiliary network of the InceptionV3 architecture (6) was weighted by a factor 0.4. The fracture detection CNNs were trained on frontal view (including AP/PA, ulnar deviated AP/PA, and oblique) and lateral view radiographs separately, as we found that this led to a better performance than training a single CNN on both views.

In all training configurations, data augmentations were applied to the mini-batches for improving the generalization performance of the CNNs. Table E3 provides an overview of the data augmentations and corresponding parameters settings used for each configuration. For the fracture detection and laterality classification CNN, the horizontal flipping and rotation augmentations were applied using the Albumentations image augmentation library (10).

**Appendix E7.** Evaluation Procedure for the Auxiliary Networks

The scaphoid localization and the laterality classification CNN were cross-validated on dataset 1 using 10 folds (no patient overlap) and were tested on dataset 2. The following metrics were calculated for evaluating the scaphoid localization CNN: sensitivity, PPV, average intersection over union (IoU), and a confusion matrix. The IoU threshold for non-maximum suppression was set to 0.45, and the confidence score and IoU threshold for detection were respectively set to 0.25 and 0.5. A scaphoid was visible on all test radiographs. For the laterality classification CNN, the accuracy and a confusion matrix were calculated.

**Appendix E8.** Scaphoid Localization and Laterality Classification Results

On dataset 1, the scaphoid localization CNN achieved a sensitivity of 99.8% (15669/15703), PPV of 99.9% (15669/15691), and mean IoU of 0.961 (SD = 0.027). On dataset 2, it achieved a sensitivity of 100% (1117/1117), PPV of 99.8% (1117/1119), and mean IoU of 0.958 (SD = 0.030). Table E4 shows an overview of the results per radiographic view on both datasets (see Table E5 and E6 for the corresponding confusion matrices). Approximately half of the undetected scaphoids in dataset 1 (8/17) were broken in largely displaced sections or were severely degenerated, while other undetected scaphoids were not fully depicted, tilted (lateral view), or not fully grown. All background detections in dataset 1 (n=5) and dataset 2 (n=2) were carpal bones (capitate, lunate, triquetrum) from young patients, except in two cases where an accessory ossicle and a round artifact were detected. The source of detected but misclassified scaphoids in dataset 1 (n=17) were oblique view radiographs, except in two cases where the radiograph was cropped towards to scaphoid.

The laterality classification CNN achieved an accuracy of 99.9% (15684/15703) on dataset 1 and an accuracy of 99.9% (1116/1117) on dataset 2 (see Table E7 and E8 for the corresponding confusion matrices). The majority of misclassified scaphoids in dataset 1 (12/19) and single misclassified scaphoid in dataset 2 were shown in radiographs with a mirrored appearance of the wrist due to the hand positioning or chosen viewpoint (i.e., AP and PA radiographs viewed from the exposure and non-exposure side respectively). Other misclassified scaphoids in dataset 1 were broken in displaced sections, not fully depicted, or tilted (lateral view).

**Appendix E9.** Scaphoid fracture detection results on Dataset 3

On dataset 3, the scaphoid fracture detection AI algorithm obtained a sensitivity of 82% (698/854, 95% CI: 79%, 84%), specificity of 82% (708/864, 95% CI: 79%, 85%), PPV of 82% (698/854, 95% CI: 80%, 84%), MLP of 77% (698/854, 95% CI: 74%, 79%), and AUC of 0.89 (95% CI: 0.87, 0.91) using all available radiographic views. The detection threshold was set to 0.608. The ROC curve with operation points and MLP curve (with 95% CI bands) is shown in Figure E3.

**Appendix E10.** ROC Curve Analysis per Radiologist

Figure E4A-E show the ROC curves with operation points and mean localization precision per radiologist with and without AI assistance on dataset 4 (65 fracture cases, 154 non-fracture cases).

**Appendix E11.** Analysis of Modified Diagnoses by Radiologists with AI Assistance

Table E9 shows the proportion of correctly and incorrectly changed scaphoid fracture diagnoses by the radiologists for the cases in dataset 4 in transition from the without AI assistance to the AI assistance condition.

**References**

1. Dutta A, Zisserman A (2019) The VIA Annotation Software for Images, Audio and Video. In: Proceedings of the 27th ACM International Conference on Multimedia, pp 2276–2279. DOI:10.1145/3343031.3350535.
2. Wong WYC, Ho PC (2011) Minimal invasive management of scaphoid fractures: From fresh to nonunion. Hand Clin 27(3):291–307.
3. Hendrix N, Scholten E, Vernhout B et al (2021) Development and Validation of a Convolutional Neural Network for Automated Detection of Scaphoid Fractures on Conventional Radiographs. Radiol Artif Intell 3(4):e200260.
4. Deng J, Dong W, Socher R, Li LJ, Li K, Fei-Fei L (2009) Imagenet: a large-scale hierarchical image database. In: 2009 IEEE conference on computer vision and pattern recognition, pp 248–255. DOI:10.1109/CVPR.2009.5206848.
5. Jocher G, Stoken A, Borovec J et al (2021) ultralytics/yolov5: v5.0 - YOLOv5-P6 1280 models, AWS, Supervise.ly and YouTube integrations. DOI:10.5281/ZENODO.4679653.
6. Szegedy C, Vanhoucke V, Ioffe S, Shlens J, Wojna Z (2016) Rethinking the inception architecture for computer vision. In: 2016 IEEE Conference on Computer Vision and Pattern Recognition, pp 2818–2826. DOI:10.1109/CVPR.2016.308.
7. Lin TY, Maire M, Belongie S, Hays J et al (2014) Microsoft coco: Common objects in context. In: European conference on computer vision, pp 740–755.
   DOI:10.1007/978-3-319-10602-1_48.
8. Sutskever I, Martens J, Dahl G, Hinton G (2013) On the importance of initialization and momentum in deep learning. In: Proceedings of the 30th International Conference on Machine Learning, in PMLR, vol 28, pp 1139–1147.
9. Kingma DP, Ba J (2015) A method for stochastic optimization. Available via https://arxiv.org/abs/1412.6980. Accessed 29 June 2022.
10. Buslaev A, Iglovikov VI, Khvedchenya E, Parinov A, Druzhinin M, Kalinin AA (2020) Albumentations: Fast and Flexible Image Augmentations. Information 11(2):125.

**Tables**

| **Table E1: Additional Imaging Parameters of the Radiographs in the Experimental Datasets** | | | |  | |
| --- | --- | --- | --- | --- | --- |
| Variable | Dataset 1 | Dataset 2 | Dataset 3 | | Dataset 4 |
| KVP | 36.26 ± 17.81 | 48.78 ± 1.03 | 33.27 ± 19.82 | | 45.34 ± 10.42 |
| Exposure (mAs) | 2.50 ± 1.42 | 2.39 ± 0.49 | 2.36 ± 1.55 | | 2.07 ± 0.61 |
| Manufacturers  x-ray devices | Siemens Healthcare  Agfa Healthcare  Canon Inc. | Philips Healthcare | Siemens Healthcare  Agfa Healthcare  Canon Inc. | | Philips Healthcare |
| Model names  x-ray devices | FD-X* (Siemens)  Fluorospot Compact FD* (Siemens)  ADC 5146/51xx* (Agfa)  ADC Compact Plus* (Agfa)  CXDI (Canon)  ADC Solo (Agfa)  DX-G (Agfa) | DigitalDiagnost*  DuraDiagnostCompact  MobileDiagnost wDR | FD-X* (Siemens)  ADC Compact Plus* (Agfa)  ADC 5146/51xx* (Agfa)  Fluorospot Compact FD* (Siemens)  ADC Solo (Agfa)  CXDI (Canon)  DX-G (Agfa) | | DigitalDiagnost*  DuraDiagnostCompact*  Essenta DR*  PCR Eleva  MobileDiagnost wDR |

Note. — The mean KVP and exposure are reported with the standard deviation. The model names of the x-ray devices are listed in descending order of frequency: the names marked with an asterisk account for 95 percent or more of the data. KVP = kilovoltage peak, mAs = milliampere-seconds.

| **Table E2: Overview of the Reference Standards of Dataset 4** | | |
| --- | --- | --- |
| Reference standard | Patients without fracture | Patients with fracture |
| Follow-up CT and MRI | 0 (0%) | 0 (0%) |
| Follow-up MRI | 2 (1%) | 1 (1%) |
| Follow-up CT | 12 (8%) | 10 (15%) |
| Follow-up scaphoid radiographs | 15 (10%) | 14 (22%) |
| Follow-up hand or wrist radiographs | 21 (15%) | 16 (25%) |
| Previous imaging studies | 27 (19%) | 6 (9%) |
| Clinical information only | 67 (47%) | 18 (28%) |
| Total | 144 (100%) | 65 (100%) |

Note. — Rounding errors were resolved using the largest remainder method.

| **Table E3: Overview of the Data Augmentations and Parameter Settings** | | | | | |
| --- | --- | --- | --- | --- | --- |
| Augmentation | Scaphoid localizer | Laterality classifier | Fracture detectors | Settings | |
|  | (SL) | (LC) | (FD) | Parameter | Value |
| Horizontal flip | ✓ |  | ✓ | probability | 50% |
| Rotate |  | ✓ | ✓ | probability | 50% (LC, FD)  100% (SL) |
|  |  |  |  | rotation range in degrees | [-20, 20] |
| Translate | ✓ |  |  | probability | 100% |
|  |  |  |  | translate fraction range | [-0.1, 0.1] |
| Scale | ✓ |  |  | probability | 100% |
|  |  |  |  | scale gain range | [-0.5, 0.5] |
| Mosaic^1^ | ✓ |  |  | probability | 100% |
| Gaussian noise |  | ✓ | ✓ | probability | 50% |
|  |  |  |  | mean | 0 |
|  |  |  |  | standard deviation | [0.01 × intensity range, 0.05 × intensity range] |

Note. — ^1^ “Mosaic” refers to a data augmentation method proposed by the author of the YOLOv5 framework (see ref 5) that combines four randomly sampled images into four tiles with various ratio’s (combined images may be cropped or zero padded).

| **Table E4. Scaphoid Localization Results** | | | | | | |
| --- | --- | --- | --- | --- | --- | --- |
|  | Sensitivity (%) | | PPV (%) | | IoU | |
| Input | Value | Proportion | Value | Proportion | Mean | SD |
| Dataset 1 |  |  |  |  |  |  |
| Any view | 99.8 | 15669/15703 | 99.9 | 15669/15691 | 0.961 | 0.027 |
| Frontal view^1^ | 99.9 | 11553/11567 | 99.9 | 11553/11569 | 0.962 | 0.023 |
| Lateral view | 99.5 | 4116/4136 | 99.9 | 4116/4122 | 0.956 | 0.034 |
| Dataset 2 |  |  |  |  |  |  |
| Any view | 100 | 1117/1117 | 99.8 | 1117/1119 | 0.958 | 0.030 |
| Frontal view^1^ | 100 | 676/676 | 100 | 676/676 | 0.967 | 0.018 |
| Lateral view | 100 | 441/441 | 99.5 | 441/443 | 0.944 | 0.039 |

Note.— Dataset 1 was used for training and validation (10-fold cross-validation results are presented in this table) and dataset 2 was used for testing. IoU = intersection over union, PPV = positive predictive value.

^1^ The frontal view category included anterior-posterior/posterior-anterior (AP/PA), ulnar-deviated AP/PA, and oblique views.

| **Table E5: Confusion Matrix of the Scaphoid Localization Results on Dataset 1 (n=15703)** | | | | |
| --- | --- | --- | --- | --- |
|  |  |  | Ground-truth |  |
|  |  | Frontal view scaphoid^1^ | Lateral view scaphoid | Background |
| AI | Frontal view scaphoid^1^ | 11553 | 14 | 2 |
|  | Lateral view scaphoid | 3 | 4116 | 3 |
|  | No detection | 11 | 6 | NA |

Note. — Each case represents one scaphoid. “Background” refers to scaphoid detections with no or insufficient overlap with a ground-truth scaphoid bounding box. Dataset 1 was used for training and validation (10-fold cross-validation results are presented in this table). NA = not applicable.

^1^ The frontal view category included anterior-posterior/posterior-anterior (AP/PA), ulnar-deviated AP/PA, and oblique views.

| **Table E6: Confusion Matrix of the Scaphoid Localization Results on Dataset 2 (n=1117)** | | | | |
| --- | --- | --- | --- | --- |
|  |  |  | Ground-truth |  |
|  |  | Frontal view scaphoid^1^ | Lateral view scaphoid | Background |
| AI | Frontal view scaphoid^1^ | 676 | 0 | 0 |
|  | Lateral view scaphoid | 0 | 441 | 2 |
|  | No detection | 0 | 0 | NA |

Note. — Each case represents one scaphoid. “Background” refers to scaphoid detections with no overlap with a ground-truth scaphoid bounding box. Dataset 2 was used for testing. NA = not applicable.

^1^ The frontal view category included anterior-posterior/posterior-anterior (AP/PA), ulnar-deviated AP/PA, and oblique views.

| **Table E7: Confusion Matrix of the Scaphoid Laterality Detection Results on Dataset 1 (n=15703)** | | | |
| --- | --- | --- | --- |
|  |  | Ground-truth | |
|  |  | Left scaphoid | Right scaphoid |
| AI | Left scaphoid | 8393 | 6 |
|  | Right scaphoid | 13 | 7291 |

Note. — Each case represents one scaphoid. Dataset 1 was used for training and validation (10-fold cross-validation results are presented in this table).

| **Table E8: Confusion Matrix of the Scaphoid Laterality Detection Results on Dataset 2 (n=1117)** | | | |
| --- | --- | --- | --- |
|  |  | Ground-truth | |
|  |  | Left scaphoid | Right scaphoid |
| AI | Left scaphoid | 579 | 0 |
|  | Right scaphoid | 1 | 537 |

Note. — Each case represents one scaphoid. Dataset 2 was used for testing.

| **Table E9: Proportion of Correctly and Incorrectly Changed Answers by the Radiologists with AI Assistance and their Correlation with the AI Answers** | | | | | | |
| --- | --- | --- | --- | --- | --- | --- |
|  | Correctly changed answers  with AI assistance (%) | | | Incorrectly changed answers  with AI assistance (%) | | |
| Reader | Value | Proportion | Pearson correlation with AI answers | Value | Proportion | Pearson correlation with AI answers |
| Rad1 | 5 | 11/219 | 0.85 | 4 | 9/219 | 0.02 |
| Rad2 | 11 | 24/219 | 0.51 | 4 | 9/219 | -0.22 |
| Rad3 | 10 | 21/219 | 0.68 | 18 | 18/219 | 0.34 |
| Rad4 | 7 | 15/219 | 0.69 | 5 | 12/219 | 0.57 |
| Rad5 | 13 | 29/219 | 0.32 | 9 | 19/219 | -0.26 |

**Figures**

**Figure E1.** Flowchart for the inclusion and exclusion of samples in datasets 1 and 3 (training data). The number of studies at each step is denoted with n. Radboudumc = Radboud University Medical Center.

^1^ all studies from the Radboud University Medical Center in the scaphoid segmentation dataset in Hendrix et al (see ref 3) were selected. ^2^ studies were excluded when the scaphoid could not be properly localized due metal osteosynthesis material, a thick cast, severe scaphoid degeneration, or insufficient image quality. ^3^ studies were excluded when the scaphoid was not mentioned in the report and when they contained a series description mentioning a cast. ^4^ exclusion due to casts, metal osteosynthesis material, excessive degeneration, underdevelopment (children), insufficient image quality, resections, and studies not indicating possible presence or absence of a scaphoid fracture or studies unrelated to bone fracture diagnosis. ^5^ in addition to the exclusion criteria in note 4, studies were excluded when the scaphoid was incompletely depicted and when no definitive fracture diagnosis could be established. ^6^ studies with a negative scaphoid fracture diagnosis were excluded when they had no follow-up between one and four weeks.


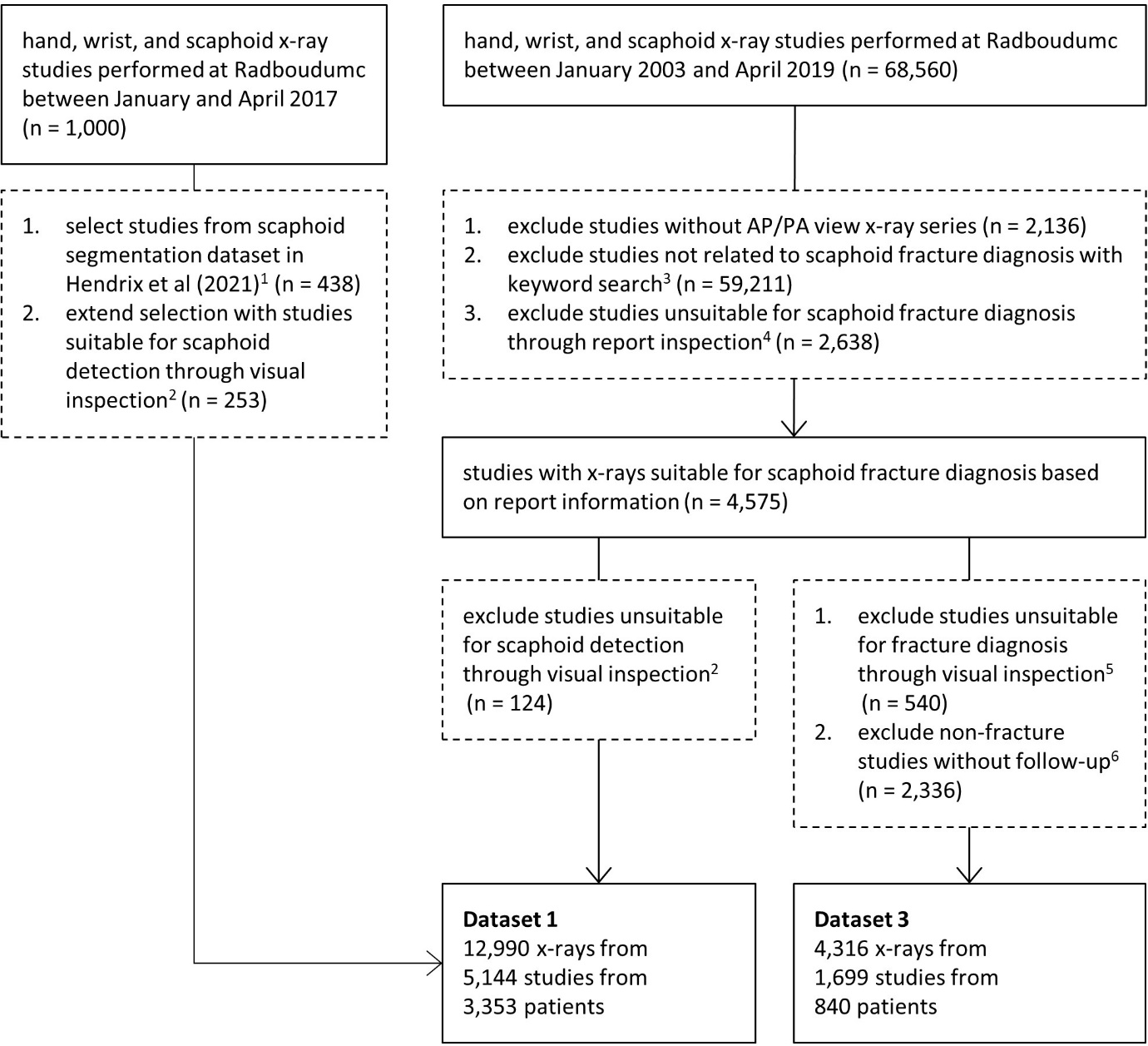


**Figure E2.** Flowchart for the inclusion and exclusion of samples in dataset 2 (test data). The number of studies at each step is denoted with n. DBC = diagnosis-treatment combination, ICD-10 = International Classification of Diseases Version 10, JBZ = Jeroen Bosch Hospital.

^1^ all studies from the JBZ in the scaphoid segmentation dataset in Hendrix et al (see ref 3) were selected. ^2^ studies were excluded when the scaphoid could not be properly localized due metal osteosynthesis material, a thick cast, severe scaphoid degeneration, or insufficient image quality.


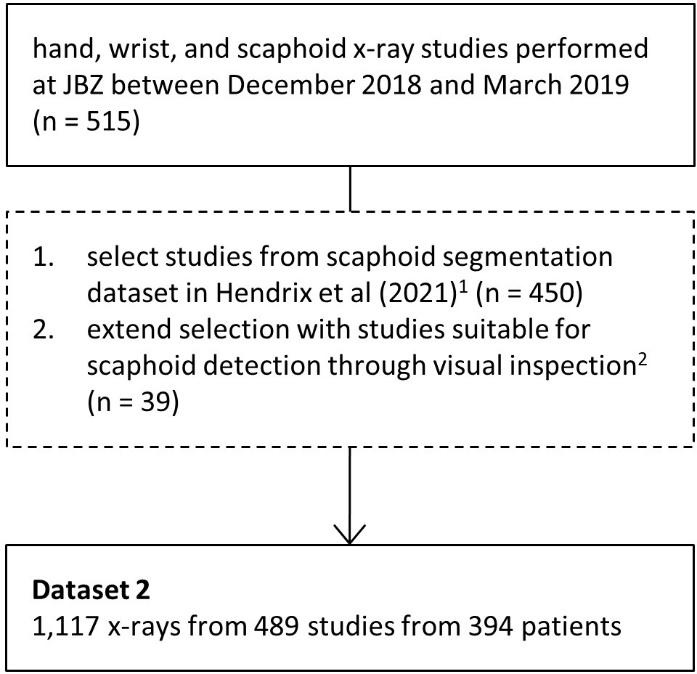


**Figure E3.** Receiver operating characteristic (ROC) curve (blue) with operating point of the automated scaphoid fracture detection results based on all available radiographic views from dataset 3 (854 fracture cases, 864 non-fracture cases; each case represents one hand from one study). The corresponding mean localization precision curve (orange) is shown as well. The shaded bands represent 95% confidence intervals. The black line represents no ability to discriminate between fracture and non-fracture cases.


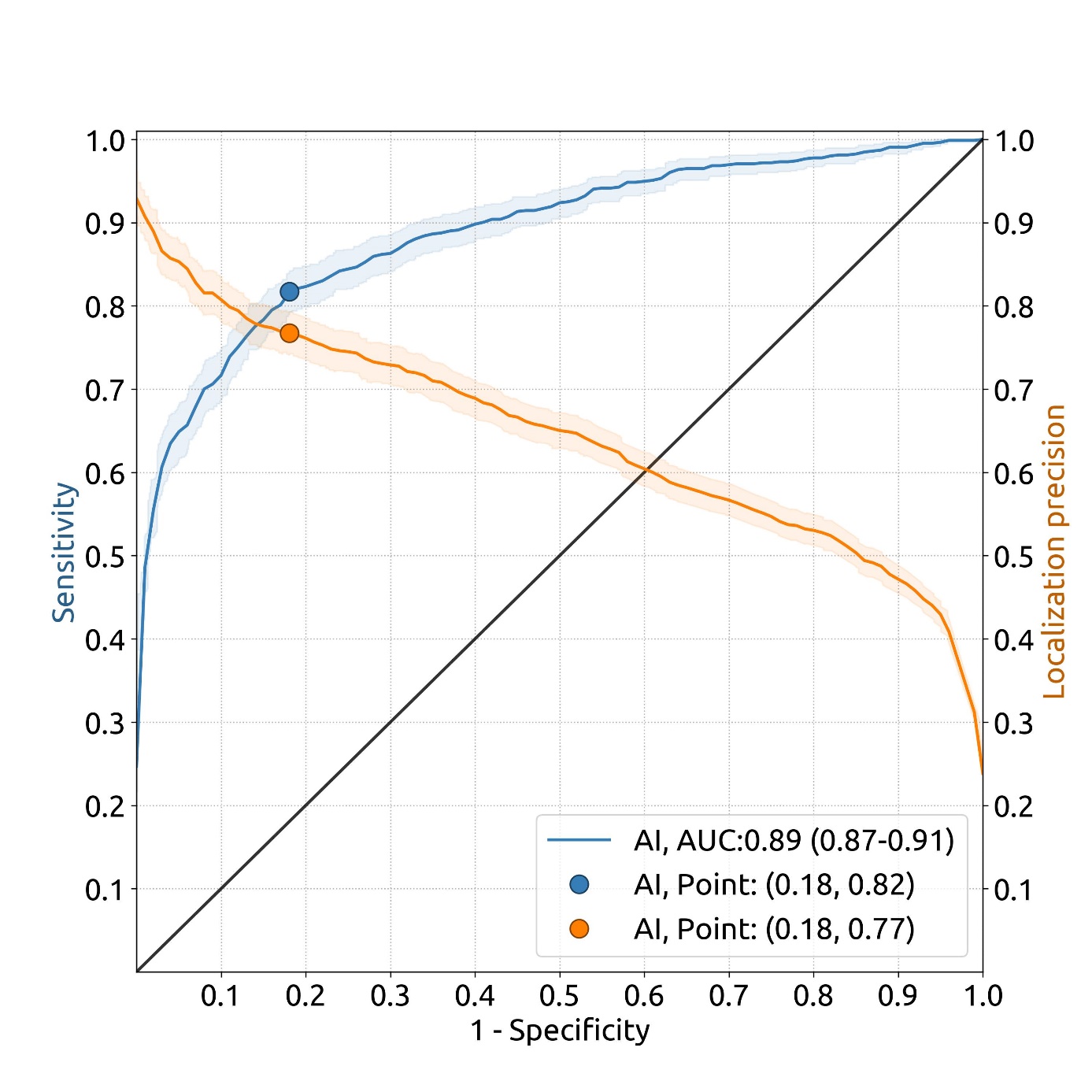


**Figure E4.** Receiver operating characteristic (ROC) curves with operating points of each radiologist (A-E) with and without artificial intelligence (AI) assistance (dashed and dense blue line) on dataset 4 (65 fracture cases, 154 non-fracture cases; each case represents one hand from one patient). The corresponding mean localization precision curves (orange) are shown as well. The shaded bands represent 95% confidence intervals. The black line represents no ability to discriminate between fracture and non-fracture cases.


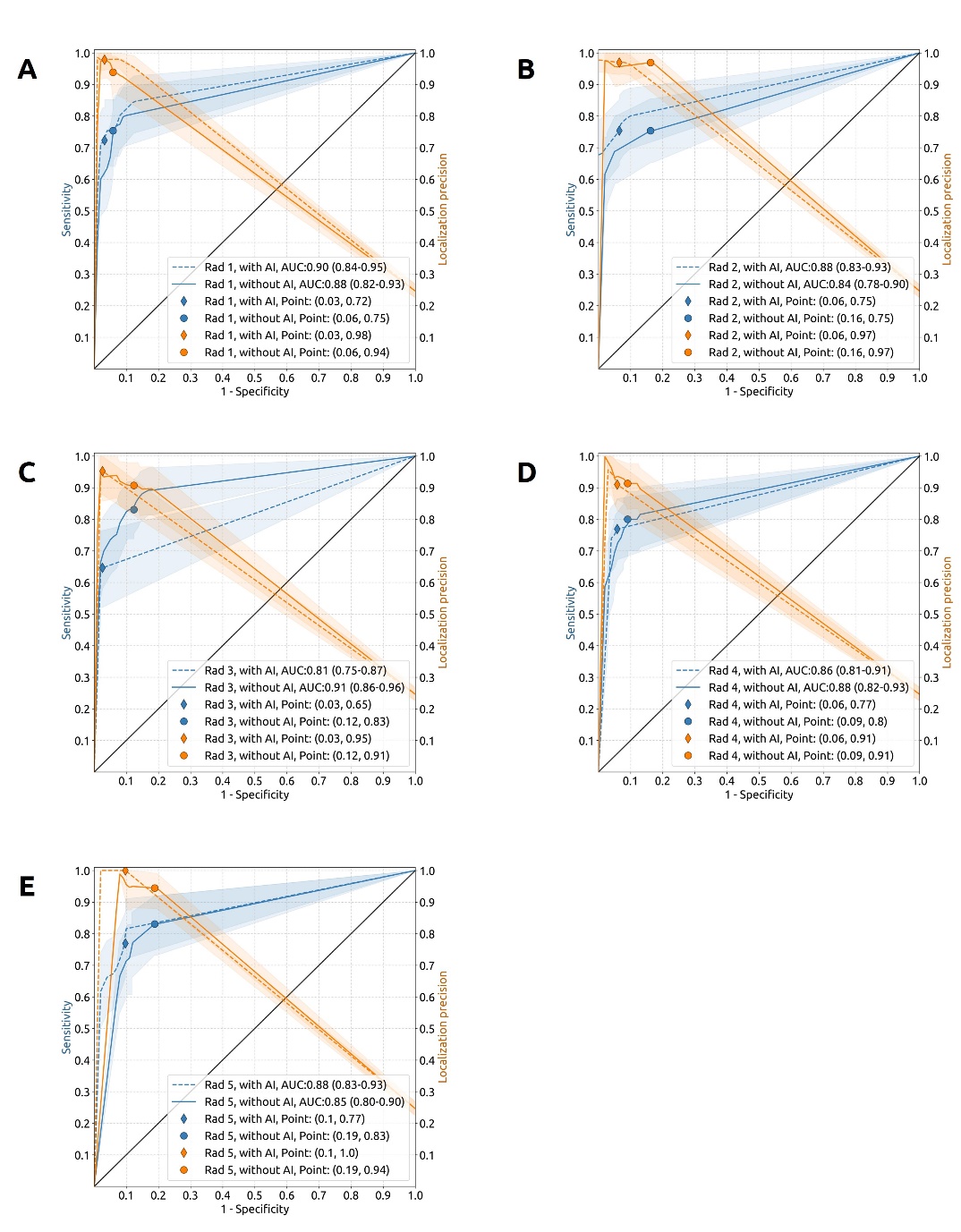

Supplement: Supplementary file 1 — (DOCX 1.16 mb) [file 330_2022_9205_MOESM1_ESM.docx]
